# Intracranial hemorrhage in term neonates

**DOI:** 10.1007/s00381-018-3788-8

**Published:** 2018-04-10

**Authors:** Hyun Sook Hong, Ji Ye Lee

**Affiliations:** Department of Radiology, Soonchunhyang University Bucheon Hospital, 170 Jomaru-ro, Wonmi-gu, Bucheon, 420-767 Republic of Korea

**Keywords:** Intracranial hemorrhage, Intraparenchymal hemorrhage, MRI, Subdural hemorrhage, Term neonates

## Abstract

**Background:**

Intracranial hemorrhage (ICH) is an uncommon but important cause of morbidity and mortality in term neonates; currently, ICH is more frequently diagnosed because of improved neuroimaging techniques.

**Purpose:**

The study aims to evaluate the clinical characteristics and neuroimaging data (pattern, size, distribution) of neonatal ICH.

**Methods:**

We reviewed MRI data from July 2004 to June 2015 for 42 term neonates with ICH who were less than 1 month old. We recorded clinical data and manifestations, mode of delivery, Apgar score at 1 and 5 min, associated hypoxic insult, birth trauma, neurological symptoms, EEG results, extent and site of hemorrhage, neurosurgical intervention, and developmental outcomes. The clinical outcome was determined for 27 neonates. Risk factors were assessed in relation to ICH.

**Results:**

A total of 42 neonates who presented with ICH underwent MR imaging 2 to 22 days postnatally (mean age 9.3 days). The majority of clinical symptoms were present in patients within the first 24 h of life (*n* = 31), but symptoms appeared until day 10 postnatally (mean 4.9 days, *n* = 11). Seizure or seizure-like activity was the most common presenting symptom (17/42, 40.5%), with apnea seen in another seven infants (7/42, 16.7%). The majority of infants had a normal prenatal course. Two patients had antenatally detected hydrocephalus. Ten had infratentorial hemorrhage, and two had supratentorial hemorrhage. A total of 30 infants had a combination of infratentorial and supratentorial hemorrhage. Subdural hemorrhage (SDH) was the most common type of hemorrhage (40/42, 95.2%), followed by nine cases of parenchymal hemorrhage, seven of subarachnoid hemorrhage, three of germinal matrix hemorrhage (GMH), and one of epidural hemorrhage (EDH). A total of 16 infants had two or more types of hemorrhage. SDH was identified along the tentorium (*n* = 38) as well as over the cerebellar hemispheres (*n* = 39), along the interhemispheric fissure (*n* = 10), and over the occipital (*n* = 13) or parietooccipital (*n* = 11) lobes. Intraparenchymal hemorrhage involved either the frontal (*n* = 4), parietal (*n* = 3), or cerebellar (*n* = 2) lobes. Traumatic delivery was suspected in 20 patients (47.6%), and perinatal asphyxia was present in 21 patients (50.0%). A low Apgar score at 5 min and a history of perinatal asphyxia were the factors that most predicted poor clinical outcomes (*n* = 12/27). Logistic regression analysis revealed that a history of perinatal asphyxia resulted in poor outcomes. No patients died. One infant required burr hole drainage of a right parietal EDH, one infant needed a subcutaneous reservoir, and three infants required a ventriculoperitoneal shunt for obstructive hydrocephalus.

**Conclusion:**

SDH was the most common type of ICH in term infants. Combined supratentorial and infratentorial hemorrhage was more common than isolated infratentorial hemorrhage in these infants. A total of 44.4% of patients had poor outcomes, with perinatal asphyxia the most common statistically significant cause.

## Introduction

Intracranial hemorrhage (ICH) in term newborns is increasingly recognized, although its true incidence and prevalence is unknown. Subdural hemorrhage (SDH), subarachnoid hemorrhage (SAH), intraparenchymal hemorrhage, and intraventricular hemorrhage (IVH) have been identified in full-term neonates [1–5]. Full-term neonates with ICH commonly present with clinical features such as apnea, bradycardia, and seizures [1–5]. The true incidence of ICH is likely higher than reported, as only a fraction of infants with ICH present with clinical features [2]. The majority of patients are managed without surgical intervention [4]. Several factors increase the risk for symptomatic ICH in full-term newborns, including prolonged, precipitous, vaginal breech, instrumental, forceps, or ventouse delivery as well as primiparity, high multiparity, and extreme fetal weight [2, 6–8].

ICH in term newborns occurs near the falx and tentorium cerebelli, producing posterior fossa hemorrhage in the dural space or within the brain parenchyma [1, 2]. Intraparenchymal hemorrhage is less frequent than SDH or SAH in term newborns [4]. The major causes of IVH are birth trauma and asphyxia [1]. Newborns rarely develop EDH, as the middle meningeal artery, which is not yet encased within the bone, is able to move freely following displacement of the skull. EDH may occur in the absence of skull fracture, such as when an external blow causes the outer layer of the dura to detach from the inner plate of the skull. This most often occurs following a difficult forceps extraction [2]. Most SDH is due to trauma. Traumatic SDH results from a rupture of veins in the subdural space, with bleeding occurring from within the venous sinus or cerebellum [5]. However, SDH in infants has a different pattern from that in older children and adults. Some reports suggest that a dural origin exists for thin film subdural bleeding in young babies [9, 10]. SDH in newborns is usually due to injuries sustained at birth and originates from either the falx or the tentorium. Hemorrhage first dissects the dural connective tissue before leaking into the subdural space [10]. SDH is a recognized finding in perinatal and pediatric autopsy. Intradural hemorrhage (IDH) at autopsy is not related to trauma in 72% of children younger than 5 months of age, and hypoxia-induced change in the permeability of the vessels is likely involved [10, 11]. ICH has also been reported in asymptomatic term infants. The prevalence was reported as 8% to 45.5% when using a 1.5-T MR imager [12–14]. These reports suggest that asymptomatic ICH in term newborns is more frequent than previously thought.

Here, we evaluate the clinical characteristics and neuroimaging data (pattern, size, distribution) of neonatal ICH.

## Materials and methods

### Patients

Our institutional review board approved this retrospective observational descriptive study of prevalence, and informed consent was waived. Between July 2004 and June 2015, 42 term neonates from 37-week gestation to 1 month of age who had ICH on MRI were reviewed.

Clinical data were recorded and included the mode of delivery, clinical manifestations, the Apgar score at 1 and 5 min, history of hypoxic insult or birth trauma, neurological symptoms, electroencephalography (EEG) results, the extent and site of hemorrhage, results of ophthalmologic examination of the retina, neurosurgical interventions, and developmental outcomes, when available.

The mode of delivery was classified as spontaneous vaginal, emergency or elective cesarean section (C/S), or assisted vaginal delivery with either forceps or vacuum. Birth weight, gestational age at birth, gestational age at MR imaging, onset of symptoms, and EEG results were reviewed. Patients with a history of perinatal asphyxia or the presence of hypoxic ischemic injury on MRI were classified as having hypoxic insult. Patients with fracture, cephalohematoma, scalp laceration, bruising associated with assisted delivery, or retinal hemorrhage were classified as having birth trauma.

### Assessment of clinical outcomes

Clinical outcomes were assessed and recorded at the clinical follow-ups from 48 months to 10 years (*n* = 24; mean 2.6 years) or using the Bayley score [15] (*n* = 3). Fifteen patients were transferred or lost to follow-up. Patients who underwent rehabilitation therapy for motor or speech impediments, or who had hypotonia, or who required a shunt operation for obstructive hydrocephalus (*n* = 24) or developmental delay as assessed by the Bayley score (*n* = 3) were classified as having a poor outcome. Correlations between risk factors and hemorrhage were calculated using the Mann–Whitney *U* test and Fisher’s exact test. Logistic regression analyses were performed for patients with poor outcomes.

### Neuroimaging

MRI was performed with a Signa HDxt 1.5-T MR imaging scanner (GE Healthcare, Milwaukee, WI, USA) using the following imaging sequences: (1) three-plane localizer; (2) axial fast spin-echo (FSE) T2-weighted imaging with a repetition time (TR)/echo time (TE) of 3500/102 ms; (3) axial fluid-attenuated inversion recovery (FLAIR) with a TR/TE of 8000/120 ms, TI of 2000 ms; (4) axial conventional SE T1WI with a TR/TE of 500/16 ms; (5) axial diffusion-weighted echo-planar imaging (DWI EPI) with a TR/TE of 8000/97.8 ms; (6) axial gradient-echo T2* imaging with a TR/TE of 450/15 ms, flip angle of 20°; (7) coronal FSE T2 imaging with a TR/TE of 3500/102 ms; and (8) sagittal SE T1 with a TR/TE of 500/9 ms.

Hemorrhage type was classified according to the site (supratentorial, infratentorial, or a combination) or compartment involved (EDH, SDH, SAH, intraparenchymal, GMH, or IVH). The brain lobe involved (frontal, temporal, parietal, and occipital), the maximum thickness of the hemorrhage, and the presence of associated brain parenchymal lesions were also recorded. In infants with SDH in multiple locations, the size of the largest SDH was recorded. The presence of cephalohematoma was also recorded.

### Statistical analysis

Data are reported as means ± standard deviations for continuous variables and as *n* (%) for categorical variables. *P* values were calculated by the Mann–Whitney *U* test for continuous variables and Fisher’s exact test for categorical variables. *P* < 0.05 was considered significant. Logistic regression analyses were performed for poor outcomes in pediatric patients with intracranial hemorrhage. All statistical analyses were performed using R (version 3.3.3; R Foundation for Statistical Computing, Vienna, Austria).

## Results

Between July 2004 and June 2015, 42 full-term neonates (22 males and 20 females) with ICH were admitted to our neonatal intensive care unit (NICU). The clinical characteristics of these neonates as well as the locations of their ICH are summarized in Table 1. There was no statistically significant difference in mean gestational age (39.2 ± 1.1 weeks), birth weight (3083.1 ± 405.0 g), mode of delivery, Apgar score at 1 and 5 min, birth history, EEG abnormalities, or initial presence of seizures with respect to the location of the hemorrhage. On full blood count evaluation, none of the neonates showed signs of anemia, thrombocytopenia, or abnormal platelet counts. Seven babies were delivered in our hospital, with the remaining 35 having been transferred to our NICU from outside hospitals.

**Table 1:** Clinical characteristics with respect to the location of intracranial hemorrhage

Variable	Total (*n* = 42)	Infratentorial (*n* = 10)	Other (*n* = 32)	*P* value
Male	22 (52.4%)	4 (40.0%)	18 (56.2%)	0.592
Age at MRI (days)	9.4 ± 6.0	9.4 ± 5.7	9.4 ± 6.1	0.965
Gestational age (weeks)	39.2 ± 1.1	39.4 ± 1.2	39.2 ± 1.1	0.556
Birth weight (g)	3083.1 ± 405.0	3087.0 ± 358.1	3081.9 ± 423.9	0.973
Delivery method				0.572
Elective cesarean section	1 (2.4%)	0 (0.0%)	1 (3.1%)	
Emergency cesarean section	9 (21.4%)	1 (10.0%)	8 (25.0%)	
Spontaneous vaginal delivery	30 (71.4%)	8 (80.0%)	22 (68.8%)	
Instrumental delivery	2 (4.8%)	1 (10.0%)	1 (3.1%)	
Apgar score at 1 min	6.7 ± 2.6	6.4 ± 2.8	6.8 ± 2.6	0.435
Apgar score at 5 min	8.0 ± 2.1	7.1 ± 2.8	8.3 ± 1.7	0.269
Birth history				
Perinatal asphyxia	21 (50.0%)	4 (40.0%)	17 (53.1%)	0.717
Traumatic delivery	20 (47.6%)	6 (60.0%)	14 (43.8%)	0.592
EEG				0.48
Not measured	11 (26.2%)	3 (30.0%)	8 (25.0%)	
Normal	16 (38.1%)	5 (50.0%)	11 (34.4%)	
Abnormal	15 (35.7%)	2 (20.0%)	13 (40.6%)	
Neurological signs				0.277
No	21 (50.0%)	7 (70.0%)	14 (43.8%)	
Yes	21 (50.0%)	3 (30.0%)	18 (56.2%)	

Data are means ± standard deviations for continuous variables and *n* (%) for categorical variables. *P* values were calculated using the Mann–Whitney *U* test for continuous variables and Fisher’s exact test for categorical variables

The majority of symptoms developed within 24 h of birth (*n* = 31), with the rest developing 2–10 days postnatally (mean 4.9 days; *n* = 11). Seizure or seizure-like activity (*n* = 17, 40.5%), apnea (*n* = 7, 16.7%), decreased activity (*n* = 3), respiratory distress (*n* = 3), jaundice (*n* = 3), cephalohematoma (*n* = 2), meconium aspiration (*n* = 2), known hydrocephalus (*n* = 2: abnormal antenatal US in one, detected on fetal MR in the other), intrauterine growth retardation (*n* = 1), skin defect with incontinentia pigmentosa (*n* = 1), and bruising (*n* = 1) were common presentations. The majority of neonates had a normal prenatal course. Two neonates had antenatally detected hydrocephalus. Infants were born by spontaneous vaginal delivery (*n* = 30), emergency C/S (*n* = 9), instrumental delivery (vacuum extraction; *n* = 2), or elective C/S delivery (*n* = 1). There were no statistically significant differences in the location of hemorrhage by clinical outcome. The majority of infants were born to primigravida mothers (*n* = 32), with seven of the neonates being second-born children and one being a third-born child. The birth order of two of the neonates was unknown.

**Fig. 1:**
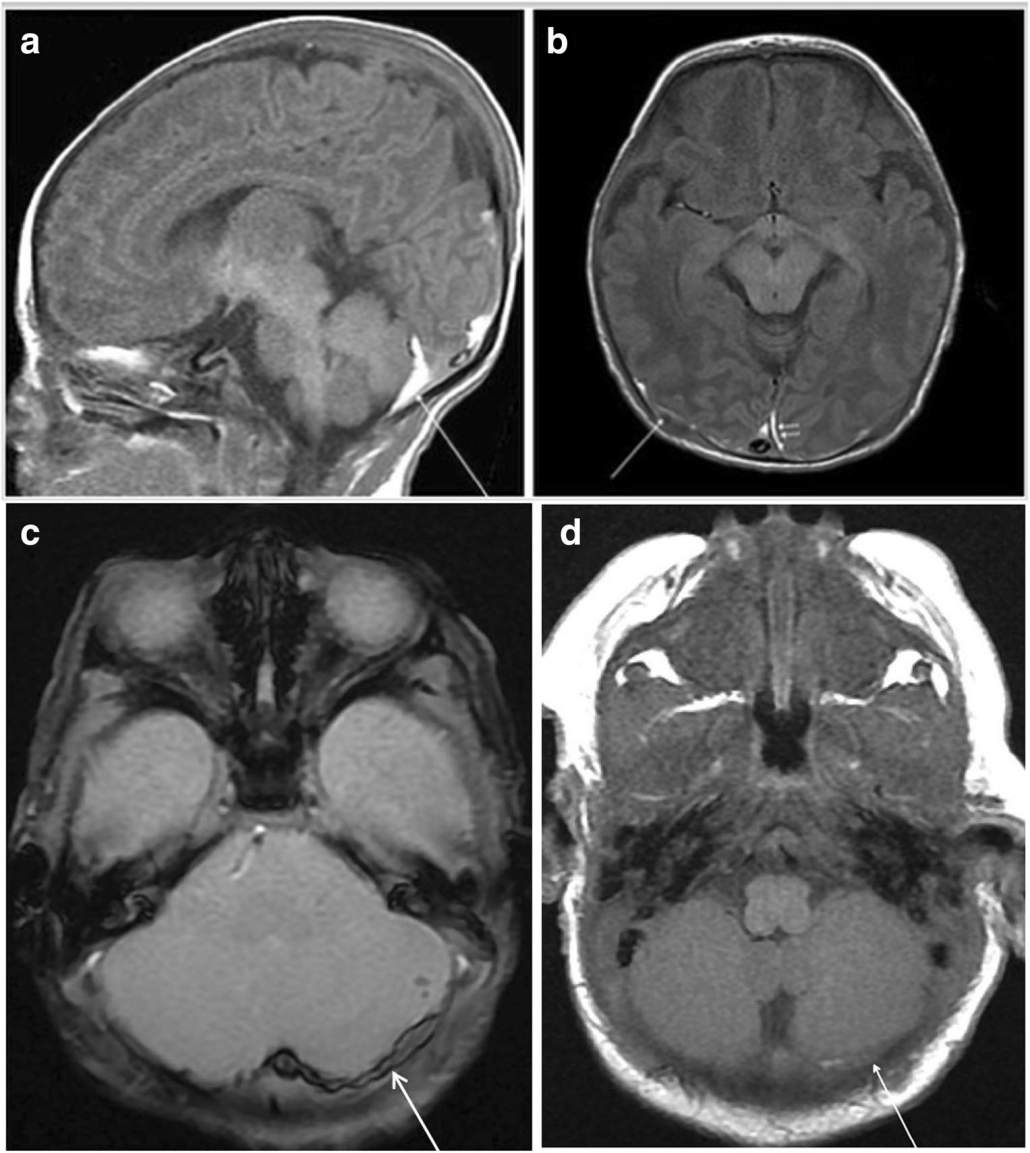
A 9-day-old male neonate exhibited supratentorial and infratentorial SDH, SAH, and intraparenchymal hemorrhage on imaging. He was born via spontaneous vaginal delivery at 38+6 weeks, weighing 2680 g. Within 24 h after birth, he developed apnea and seizures. Apgar scores at 1 and 5 min were 3 and 5, respectively. He was born to a primigravida with a history of prolonged labor. Ophthalmologic examination revealed the development of retinal hemorrhage in both eyes. His Bayley scale score at 8 months showed mild developmental delay. **a** Sagittal T1WI shows combined supratentorial and infratentorial SDH (arrow). **b** Axial T1WI imaging demonstrates a high-signal SDH over both parietooccipital lobes (arrow), and along the posterior interhemispheric fissure (arrowheads). **c** GE axial imaging shows puntate intraparenchymal hemorrhage of the left cerebellar hemisphere as well as SDH over the left cerebellar hemisphere (arrow). **d** SDH (arrow) had nearly resolved at the 1-month follow-up MR

MR imaging was performed 2–22 days postnatally (mean age at MR imaging 9.3 days). The involved compartments of ICH are summarized in Table 2. Ten term infants had infratentorial hemorrhage, whereas two had hemorrhage in the supratentorial region. A total of 30 neonates had a combination of both infratentorial and supratentorial hemorrhage (Fig. 1).

**Table 2:** Characteristics of the intracranial hemorrhage of term neonates

Site	Infratentorial	Supratentorial	Supra + infra	Total
Involved compartment				
EDH	0		1	1^a^
SDH	10	2	28	40^a^
SAH	0		7^a^	7^a^
Parenchymal	2^b^		7^a^	9^a^
GMH	0		3^a^	3^a^
Poor outcome	2		10	12

Data are reported as *n* = number of patients

^a^A total of 16 infants had two or more types of hemorrhage (SDH + SAH + intraparenchymal: *n* = 4, SDH + SAH: *n* = 3, SDH + GMH: *n* = 3, SDH + intraparenchymal: *n* = 5, EDH + SDH: *n* = 1). SDH was located along the tentorium (*n* = 38, range 1.1–5.48 mm, mean maximum thickness 2.3 mm); over the cerebellar hemispheres (*n* = 39, 0.9–7.29 mm, mean 2.95 mm); along the interhemispheric fissure (*n* = 10); over the occipital lobes (*n* = 13; range 0.9–2.84, mean 1.72 mm); over the parietooccipital lobes (*n* = 11, range 1.56–5.41, mean 2.71 mm); and in the frontoparietal (*n* = 3), temporoparietooccipital (*n* = 3), and parietal (*n* = 4) regions

^b^Among infants with intraparenchymal hemorrhage, the frontal (*n* = 4, 4.4 × 1.1–2.0 × 8.5 mm), parietal (*n* = 3, 1 × 1–12.3 × 13.85 mm), and cerebellar (*n* = 2, 9.0–4.0 mm) lobes were most commonly involved. Follow-up imaging was completed on 9/42 patients (21.4%) with SDH from 20 days to 7 months postnatally. SDH had resolved or decreased at follow-up in all patients

**Fig. 2:**
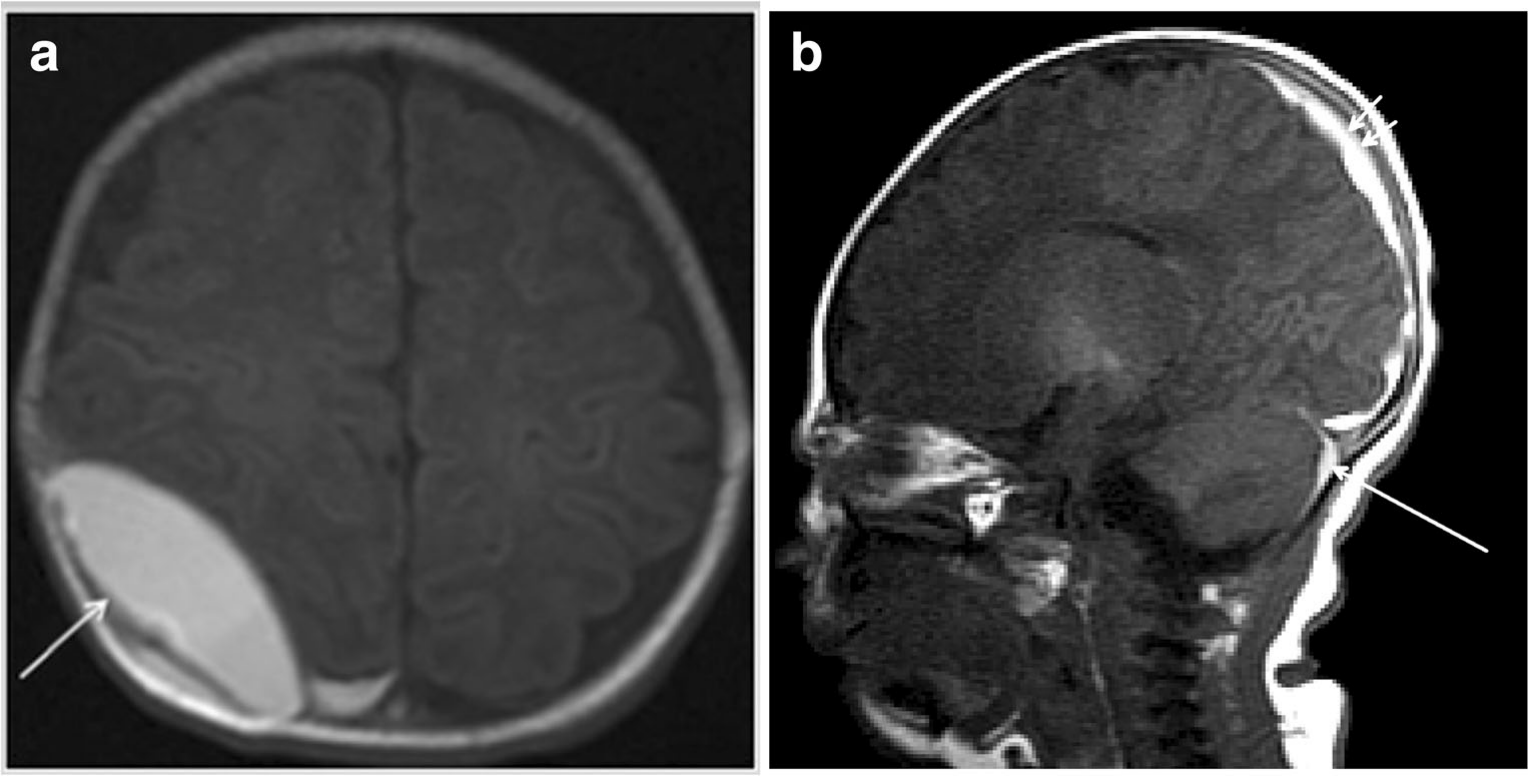
A 9-day-old female neonate exhibited both supratentorial and infratentorial SDH and EDH at the right parietal convexity. She was born via spontaneous vaginal delivery at 38 weeks weighing 3070 g. She presented with jaundice 4 days postnatally and exhibited **a** EDH (arrow) at the right parietal convexity on axial T1WI MR imaging and **b** SDH over both cerebellar and parietooccipital lobes (short arrows). She underwent burr hole drainage of EDH 11 days postnatally. She was followed in the clinic for 4 years and developed normally

**Fig. 3:**
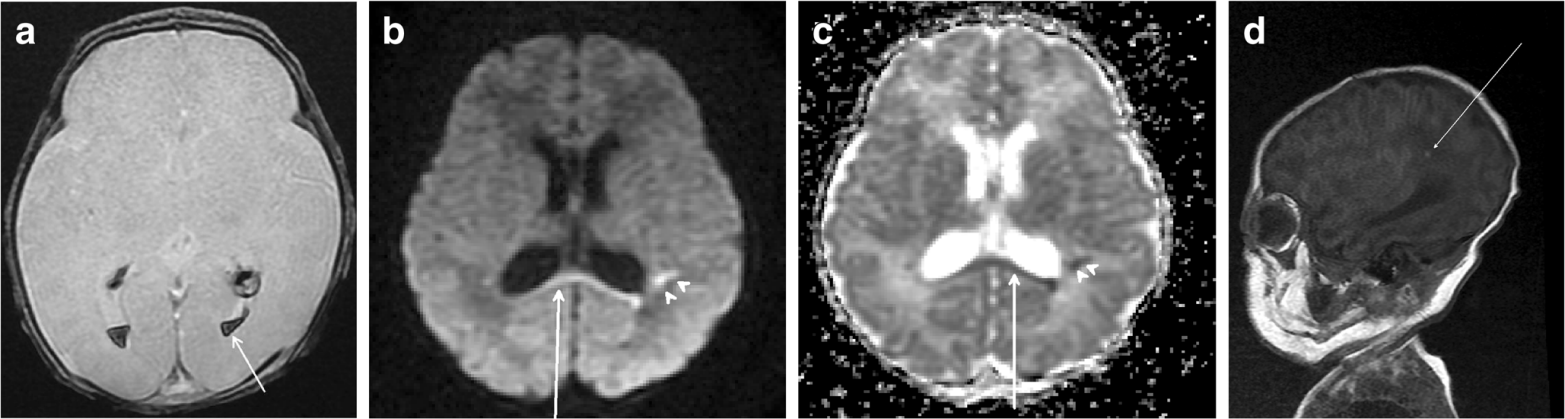
A 13-day-old female neonate was transferred to our hospital because of a seizure. She was delivered by an elective cesarean section and had a PDA and ASD. Apgar scores at 1 and 5 min were 8 and 10, respectively. MRI obtained 16 days postnatally showed **a** bilateral intraventricular hemorrhage on axial GE imaging and **b**, **c** diffusion restriction of the corpus callosum (arrow) and high-signal foci in both frontoparietal periventricular white matters (arrowheads) on axial DW and ADC map. **d** Several high-signal foci were observed in the left periventricular white matter on T1 sagittal scan (arrow). Bayley scale evaluation at 8 months showed normal development

SDH was the most common type of hemorrhage (40/42, 95.2%). Seven SAH, one EDH (Fig. 2), three GMH (Fig. 3), and nine intraparenchymal hemorrhages were identified. A total of 16 infants had two or more types of hemorrhage. SDH was commonly located along the tentorium (*n* = 38), over the cerebellar hemispheres (*n* = 39), along the interhemispheric fissure (*n* = 10), over the occipital lobes (*n* = 13), and parietooccipital lobes (*n* = 11). The frontal (*n* = 4), parietal (*n* = 3), and cerebellar (*n* = 2) lobes were most commonly involved in patients with intraparenchymal hemorrhage. A single lobe was involved in eight of nine infants with intraparenchymal hemorrhage. There was no significant correlation between the clinical outcome and site of hemorrhage. SDH was resolved or decreased in follow-up patients (9/42, 21.4%).

Traumatic delivery was suspected in 20 newborns (47.6%), and perinatal asphyxia was suspected in 21 newborns (50.0%). A low Apgar score at 5 min (*P* = 0.014) and a history of perinatal asphyxia (*P* = 0.027) were associated with poor clinical outcomes (*n* = 12/27). A low Apgar score at 1 min was not statistically significant but showed a modest trend (Table 3). A history of perinatal asphyxia was associated with poor outcomes in univariate logistic regression analyses and was statistically significant (*P* = 0.015; Table 4).

**Table 3:** Clinical characteristics by clinical outcome

Variable	Total (*n* = 27)	Good outcome (*n* = 15)	Poor outcome (*n* = 12)	*P* value
Male	15 (55.6%)	6 (40.0%)	9 (75.0%)	0.153
Age at MRI (days)	10.6 ± 6.4	10.7 ± 5.7	10.4 ± 7.5	0.66
Gestational age (weeks)	39.2 ± 1.1	39.0 ± 1.2	39.4 ± 1.0	0.404
Birth weight (g)	3042.6 ± 412.1	3013.3 ± 437.2	3079.2 ± 394.4	0.688
Delivery method				0.191
Elective cesarean section	1 (3.7%)	1 (6.7%)	0 (0.0%)	
Emergency cesarean section	7 (25.9%)	2 (13.3%)	5 (41.7%)	
Spontaneous vaginal delivery	19 (70.4%)	12 (80.0%)	7 (58.3%)	
Apgar score at 1 min	6.9 ± 2.5	7.9 ± 1.4	5.7 ± 3.1	0.055
Apgar score at 5 min	8.1 ± 2.2	9.1 ± 1.2	7.0 ± 2.7	*0.014*
Birth history				
Perinatal asphyxia	15 (55.6%)	5 (33.3%)	10 (83.3%)	*0.027*
Traumatic delivery	13 (48.1%)	6 (40.0%)	7 (58.3%)	0.576
EEG				0.212
Not measured	8 (29.6%)	6 (40.0%)	2 (16.7%)	
Normal	10 (37.0%)	6 (40.0%)	4 (33.3%)	
Abnormal	9 (33.3%)	3 (20.0%)	6 (50.0%)	
Location of intracranial hemorrhage				0.877
Infra	6 (22.2%)	4 (26.7%)	2 (16.7%)	
Other	21 (77.8%)	11 (73.3%)	10 (83.3%)	
Neurological signs				0.322
No	13 (48.1%)	9 (60.0%)	4 (33.3%)	
Yes	14 (51.9%)	6 (40.0%)	8 (66.7%)	

Data are means ± standard deviations for continuous variables and *n* (%) for categorical variables. *P* values were calculated using the Mann–Whitney *U* test for continuous variables and Fisher’s exact test for categorical variables

Italics are statistically significant

**Table 4:** Logistic regression analyses of poor outcomes in pediatric patients with intracranial hemorrhage

Variable	Univariate	
	OR (95% CI)	*P* value
Male	4.5 (0.91–27.39)	0.077
Age at MRI (days)	0.99 (0.87–1.12)	0.897
Gestational age (weeks)	1.36 (0.69–2.86)	0.389
Birth weight (g)	1 (1–1)	0.676
Delivery method		
Elective cesarean section	1 (reference)	
Emergency cesarean section	39,128,401.96 (0–NA)	0.994
Spontaneous vaginal delivery	9,129,960.46 (0–NA)	0.995
Apgar score at 1 min	0.63 (0.34–0.95)	0.066
Apgar score at 5 min	0.48 (0.18–0.88)	0.061
Birth history		
Perinatal asphyxia	10 (1.8–83.96)	0.015
Traumatic delivery	2.1 (0.46–10.41)	0.346
EEG		
Not measured	1 (reference)	
Normal	2 (0.27–18.77)	0.505
Abnormal	6 (0.81–63.13)	0.097
Location of intracranial hemorrhage		
Infra	1 (reference)	
Other	1.82 (0.29–15.25)	0.538
Neurological signs		
No	1 (reference)	
Yes	3 (0.64–15.94)	0.174

*OR* odds ratio, *CI* confidence interval

No patients in the study died, and only 5/42 (11.9%) required surgical intervention. One infant required burr hole drainage for a right parietal EDH, one needed a subcutaneous reservoir, and three (7.1%) required a ventriculoperitoneal shunt due to obstructive hydrocephalus.

## Discussion

### Clinical characteristics and outcome

Several risk factors have been reported in term newborns with ICH, but very few studies have demonstrated a relationship between these risk factors and ICH, and these often comprise only a small number of cases [3, 4, 10, 14, 16–19]. The risk of SDH and other types of hemorrhage found on imaging in symptomatic infants varies with the method of delivery. Towner et al. reported that forceps assistance, ventouse extraction, and C/S were all associated with an increased risk of ICH [6]. Towner et al. reported that successful vaginal delivery using either vacuum extraction or forceps appeared to carry no excessive risk of ICH compared with C/S. The excessive morbidity was postulated to be due to labor [6]. Benedetti stated that if an attempt at vaginal delivery fails, the risk of injury is increased regardless of the chosen method of delivery [7]. In a study by Whitby et al., those delivered by forceps after an attempted ventouse delivery were more likely to have SDH than those delivered by any other method [12]. Thus, the most common risk factor of ICH is complicated labor [6–8]. In our study, 71.4% of neonates born via spontaneous vaginal delivery and any other delivery method did not show any association with the outcome of or site of hemorrhage. A history of traumatic delivery was seen in 47.6% of patients in our study and was not associated with a poor outcome. Nulliparous women were more likely to deliver with ventouse or forceps assistance; therefore, children are at an increased risk [12, 13]. In our study, 32 mothers (76.2%) were nulliparous. Eight women were multiparous (second child *n* = 7, third child *n* = 1), an associated factor mentioned in other studies but not adequately documented.

A postmortem study of children who died of natural causes revealed an association between IDH or SDH and hypoxia. The highest incidence was seen in the perinatal period [10]. IDH and SDH were more prominent in the posterior falx and tentorium, both of which are anatomically related to an extensive intradural venous plexus [10]. Among cases with diffuse IDH on histology, 14/17 (82.4%) showed hypoxemic injury. Brouwer et al. reported a high mortality rate of 24.5% but normal neurodevelopmental outcomes in most of their surviving patients (83.8%) [3]. This high mortality rate could partially be explained by an associated high rate of perinatal asphyxia. In our patient group, there were no deaths, but 12/27 (44.4%) had a poor outcome. Infants with poor outcomes had a significantly lower Apgar score at 5 min, indicating the likelihood of perinatal asphyxia. Perinatal asphyxia was the only statistically significant risk factor in patients with poor outcomes. Hypoxia induces cerebral microvascular changes and leads to increased permeability at tight junctions [9, 10, 20]. Hypoxia coupled with increased intracranial and intravascular pressure can cause blood to leak into the extravascular compartment, resulting in IDH [11].

Thrombocytopenia is the most common laboratory condition presenting as ICH in term newborns [3, 17]. Jhawar et al. concluded that thrombocytopenia is the most important predictor of ICH and is associated with the most severe type of hemorrhage [3]. Therefore, it is recommended that a coagulation profile be obtained from all infants suspected of having ICH. Because this was a retrospective study, we had limited data on the coagulation status of our infants. However, no patients in our study presented with low platelets or anemia; therefore, additional coagulation studies were not performed.

Term newborn IVH is a rare event because it originates from the choroid plexus; however, it may occur as a result of sinovenous thrombosis [19]. Wu et al. reported an incidence of 1.2% for IVH secondary to sinovenous thrombosis [19]. In our study, no patients had isolated IVH; in the two patients who presented with IVH, it was associated with GMH.

Barring a serious initial clinical presentation, term infants with ICH tend to improve conservatively. The majority of full-term infants with intraparenchymal hematoma recover without surgical intervention, and a shunt is rarely required. In our study, 5/42 (11.9%) infants had surgery, and 3/5 (7.1%) had VP shunts installed because of obstructive hydrocephalus.

### Site and involved compartment of ICH

Prior studies have shown that infratentorial SDH is the most common subtype [3–5, 12–14, 21]. In our study, combined supratentorial and infratentorial hemorrhage was more common than an isolated infratentorial hemorrhage, with only two neonates (4.7%) presenting with supratentorial hemorrhage. SDH was most frequently seen along the tentorium (38/42, 90.5%) or above the cerebellum (39/42, 92.9%). Infratentorial SDH was also the most frequent ICH in a group of asymptomatic term newborns [12–14, 21]. With regard to SDH presenting in a supratentorial location, hemorrhage over the occipital lobe (13/42, 30.9%), over the parietooccipital lobe (11/42, 26.2%), or in the interhemispheric regions (10/42, 23.8%) was most common.

SDH in the perinatal period can follow a traumatic lesion in full-term infants and is typically found over the cerebral convexity [1]. SDH near the superior convexities of the cerebral hemispheres is not an unusual finding in intrauterine or perinatal deaths [22, 23]. In these cases, SDH more commonly presents as a thin film on the occipital convexity of the cerebral hemispheres or as a small intratentorial bleed. SDH related to birth trauma may be secondary to the tearing of the tentorium of the falx and/or to the tearing of bridging blood vessels and dural sinuses during labor [1, 11–14, 24, 25]. Increased circumferential pressure, as well as squeezing of the head in the birth canal during vaginal delivery, results in overlapping at the cranial sutures, mechanical compression, and shearing of the bridging veins, resulting in SDH. However, these rationales were recently challenged, as there is frequently an absence of ruptured bridging veins on autopsy [20]. Forensic studies suggest that vessels intrinsic to the dura may be a source of thin film subdural bleeding in young babies [9, 10]. IDH either is more prominent or is only present in the posterior falx and tentorium. IDH often starts as blood leaking from dural vascular channels, which if diffuse can occupy the entire dural thickness and eventually become SDH. The fact that IDH was most prominent or only present in the posterior falx and tentorium is explained by the presence of extensive venous plexuses in the posterior third of the falx at its inferior edge [26]. These plexuses are more prominent in neonates and regress in the first year of life [27]. This may explain the prevalence of diffuse IDH and SDH and the common intratentorial location seen in younger patients.

The annual incidence of SDH in children younger than 2 years is 12.8/100,000, and SDH is often associated with head trauma [16]. Birth-related ICH, particularly SDH, is important in the evaluation of abusive head trauma. SDH after delivery occurs in 8–50% of asymptomatic neonates [12–14, 21]. Geddes et al. [11] found IDH at autopsy not related to trauma in 72% of children younger than 5 months of age. Most of the SDHs were resolved by 4 weeks [12] and 3 months [14]. Whitby et al. also found that their nine patients with SDH first seen within 48 h of life were resolved on MR imaging at the 4-week follow-up. Rooks et al. suggested that SDH in an infant older than 3 months of age is unlikely to be birth-related regardless of the mode of delivery [14]. Therefore, patient age at the time of MR imaging may be important in determining the etiology of neonate SDH.

The present study had certain limitations. First, it lacked a control neonatal group, as normal term babies are unable to undergo MR imaging. Second, the number of patients followed up for developmental outcomes was small, and the follow-up period was relatively short. Clinical follow-up should be continued until school age, as these infants are likely to be at an increased risk for cognitive or behavioral problems. Third, the study focused on a period of 10 years, during which the standard of neonatal care improved. Fourth, follow-up imaging was only obtained in nine cases from 20 days to 4 years postnatally. Although none of our infants presented clinically with evidence of SDH rebleed, the subclinical incidence of rebleeding was not studied. All infants who were reimaged showed a complete resolution or a decrease in size of their SDH. Normal development on clinical examination is reassuring and indicates that major rebleeding did not take place.

## Conclusion

SDH was the most common type of ICH in term infants. Combined supratentorial and infratentorial hemorrhage was more common than isolated infratentorial hemorrhage. Posterior fossa involvement along the tentorium and over the cerebellum was most common. A total of 44.4% of patients had a poor outcome, with a history of perinatal asphyxia the most statistically significant factor predictive of a poor outcome.
